# Supplementary material for: Cloud BioLinux: pre-configured and on-demand bioinformatics computing for the genomics community
Source: BMC Bioinformatics. 2012 Mar 19;13:42. doi: 10.1186/1471-2105-13-42 (PMC3372431; doi:10.1186/1471-2105-13-42)
Supplement: Additional file 1 — Supplementary 1 Cloud BioLinux software documentation in the form of a mini, self-contained website. Users need to download and uncompress the .zip file, and open through a web browser the "index.html" file available on the main directory. (ZIP 1823 kb). [file 1471-2105-13-42-S1.ZIP › Cloud-BioLinux-Package-Documentation/docs/uclust.html]

Bio-Linux Software Documentation Pages

Back to search form

## uclust

|  |  |
| --- | --- |
| Name | uclust |
| Description | **uclust** is a a clustering, alignment and search algorithm capable of handling millions of sequences.  **uclust** is distributed for Bio-Linux as part of the bio-linux-qiime package. This is an old version of uclust, which can run on 64 bit systems and is available free of charge for academic use. If you are interested in using uclust outside qiime, or its sister program, usearch, we recommend you use a more up-to-date version, for which you will need a license even for academic use on 64 bit systems. Please refer to the uclust homepage.  Suggested applications of uclust include:   - Generating non-redundant protein and nucleotide sequence databases.- OTU-picking by clustering 16S/18S rRNA sequences.- Clustering reads from meta-genomic and environmental sequencing studies.- Taxonomy assignment of sequences to curated databases such as Greengenes, RDP and SILVA.- Phylogenetic analysis of very large sequence sets. |
| Homepage | http://www.drive5.com/usearch/ |
| Remote Documentation |  |
